# Conductance-based reversal potentials in spiking recurrent neural networks enhance energy efficiency and task performance

**DOI:** 10.3389/fncom.2026.1870593

**Published:** 2026-07-30

**Authors:** Miguel Rodrigues, Carmen Gasco-Galvez, Martin Vinck

**Affiliations:** 1 Donders Centre for Neuroscience, Department of Neurophysics, Radboud University Nijmegen, Nijmegen, Netherlands; 2 Ernst Strüngmann Institute (ESI) for Neuroscience in Cooperation with Max Planck Society, Frankfurt am Main, Germany

**Keywords:** biologically plausibility, conductance models, Dale's law, energy efficiency, neuron model, reversal potentials, spiking recurrent neural networks

## Abstract

Spiking recurrent neural networks (SRNNs) rival gated recurrent neural networks (RNNs) on various tasks, yet they still lack several hallmarks of biological neural networks. We introduce a biologically grounded SRNN that implements Dale's law with conductance-based stands for a-amino-3-hydroxy-5-methyl-4-isoxazolepropionic acid (AMPA) and gamma-aminobutyric acid (GABA) reversal potentials. These reversal potentials modulate synaptic gain as a function of the postsynaptic membrane potential, and we derive theoretically how they make each neuron's effective dynamics and subthreshold resonance input-dependent. We trained SRNNs on the Spiking Heidelberg Digits (SHD) dataset and show that SRNNs with reversal potentials reduce spike energy by up to 3 × , while maintaining, or increasing, task accuracy. This leads to high-performing Dalean SRNNs that substantially improve on Dalean networks without reversal potentials. SRNNs with reversal potentials exhibited spike-train statistics closer to Poisson statistics, similar to biological neurons, and showed a substantial reduction in oscillatory activity, leading to increased heterogeneity in response properties. Thus, Dale's law with reversal potentials, a core feature of biological neural networks, can render SRNNs more accurate and energy-efficient.

## 1 Introduction

Spiking recurrent neural networks (SRNNs) are a direct analog of biological neural networks (Maass, 1997) and can achieve relatively good performance on complex tasks as compared to classic RNN architectures (gated recurrent units, long short-term memory) or feedforward artificial neural networks (ANNs) (Bellec et al., 2018; Yin et al., 2021; Fang et al., 2021). SRNNs also offer energy-efficient solutions for neuromorphic computing (Roy et al., 2019). The performance of SRNNs has been improved due to several developments, including: the use of surrogate gradients to implement back-propagation-through-time (BPTT) (Werbos, 2002); the use of spiking neurons with adaptation mechanisms (Bellec et al., 2018); and increasing or optimizing the heterogeneity across neurons (Perez-Nieves et al., 2021; Bittar and Garner, 2022). However, many properties of biological neurons have yet to be implemented in SRNNs, and the ways in which these properties affect their performance and energy efficiency remain partially unexplored.

Several studies indicate that the performance of SRNNs can be significantly boosted by using neurons with adaptive thresholds or currents, as compared to the standard LIF models (Bellec et al., 2020, 2018; Baronig et al., 2025; Bittar and Garner, 2022). Networks with adaptive Linear Integrate Fire neurons (adapLIFs) are able to reproduce both subthreshold behaviors of so-called excitability class 1 and class 2 neurons (Baronig et al., 2025; Hodgkin, 1948; Izhikevich, 2000), while LIF neurons are restricted to class 1 neurons. Furthermore, these neurons exhibit resonant properties that give rise to subthreshold oscillatory dynamics, and they exhibit longer memory time constants, allowing them to solve tasks with greater memory requirements. The addition of such neurons can be seen as an example of boosting performance by introducing heterogeneity (Perez-Nieves et al., 2021).

However, the implementation of adapLIF neurons in a task-solving SRNN (Bittar and Garner, 2022; Baronig et al., 2025) still leads to voltage dynamics with major fluctuations that are not biologically plausible. A recent study suggests that clipping a neuron's voltage distribution can improve the performance of a feedforward SNN (Guo et al., 2022). In biological neurons, voltage dynamics are constrained by specific mechanisms, namely the existence of reversal potentials. Reversal potentials give rise to voltage-dependent synaptic currents and can, in fact, reverse the sign of GABAergic (pertaining to the neurotransmitter gamma-aminobutyric acid [GABA]) and glutamatergic synaptic inputs. For example, GABAergic inputs become excitatory when the receiving neuron's voltage is below the chloride reversal potential. Here, we define a new neuron model in which GABAergic and glutamatergic neurons give rise to recurrent synaptic inputs that are governed by a reversal potential. The SRNN thus contains two classes of neurons, each with positive and negative reversal potentials, mimicking Dale's law (i.e., neurons that use a single neurotransmitter), and is either inhibitory or excitatory depending on the receiving neuron's membrane potential.

We performed simulations on the benchmark Spiking Heidelberg Digits (SHD) (Cramer et al., 2022) classification task and evaluated our biologically inspired model, comparing it to the state-of-the-art neuron model adapLIF. We provide a theoretical analysis of the model's dynamics and an analysis of the subthreshold resonance of adapLIF neurons. We show that introducing a reversal potential boosts the SRNN's task performance and energy efficiency.

## 2 Results

Our starting point is the well-known adapLIF neuron model (Baronig et al., 2025; Bittar and Garner, 2022; Bellec et al., 2018), which we defined in our *baseline model*. We used a symplectic-Euler (SE) discretization adapted from Baronig et al. (2025), which is described in “Methods” section,


$$\begin{array}{ll} h_{t} & =\alpha h_{t-1}+\left(1-\alpha \right)\left(-w_{t-1}+I_{t}\right)-V_{threshold}S_{t-1}\\ S_{t} & =H\left(h_{t}-V_{threshold}\right)\\ w_{t} & =\beta w_{t-1}+ah_{t}+bS_{T}\\ I_{j}^{t} & =\overset{n}{\underset{i}{\sum }}W_{ji}^{in}x_{i}^{t}+\underset{j\neq i}{\sum }W_{ji}S_{i}^{t-1} \end{array}$$

**Figure 1:**
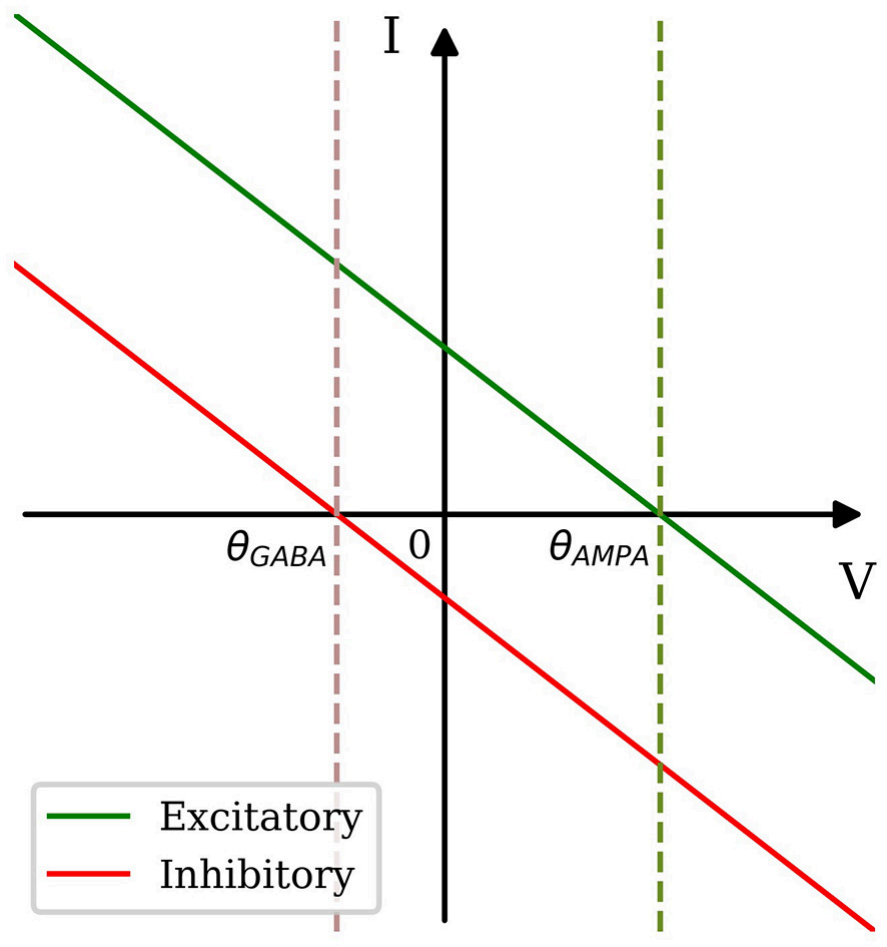
Input gains, or driving force, for excitatory (green) and inhibitory (red) pre-synaptic signals based on the post-synaptic neuron's membrane potential. θ represents the threshold for the respective glutamergic/GABAergic voltage reversal potential.

Note that using the bounds on the parameters α, β, *a*and*b* as discussed in Bittar and Garner (2022), the trained SRNN contained neurons whose membrane potentials were hypersensitive to the first external input value and diverge to either positive or negative infinities. We prevented this effect by using the bound *a*≥0 as discussed in Baronig et al. (2025); note that this bound is also required for neurons to develop subthreshold resonance (see “Methods” section). We compared the *baseline model* with two other models in which aspects of biological networks/neurons were introduced:

- *Dale model*, which uses the same equations above but with a restriction on the recurrence matrices to obey Dale's law;
- *AM+GA models*, which use Dale's law and in addition implement the voltage reversal potentials, such that synaptic inputs are modulated based on the post-synaptic neuron's membrane potential (Figure 1).

The voltage reversal potentials were mathematically described by changing the input current, ***I***, in the *baseline model* to:


$$\begin{array}{l} I_{j}^{t}=\overset{n}{\underset{i}{\sum }}W_{ji}^{in}x_{i}^{t}+\overset{n_{exc}}{\underset{i\neq j}{\sum }}W_{ji}\left(\theta_{exc}-h_{j}^{t-1}\right)S_{i}^{t-1}\\ \;+\overset{n_{inh}}{\underset{i=n_{exc}+1\neq j}{\sum }}W_{ji}\left(h_{j}^{t-1}-\theta_{inh}\right)S_{i}^{t-1} \end{array}$$

Here, θ_*exc*_ and θ_*inh*_ are the reversal potentials analogous to glutamatergic (AMPA) and GABAergic inputs, respectively. To determine the relative contributions of conductance-like gain that these “glutamatergic” and “GABAergic” reversal potentials cause, we further defined two additional models: *AMPA, GABA*, where we implemented the voltage reversal potential either for glutamatergic or GABAergic inputs (i.e., only the green/red line in Figure 1). We have set these threshold values at −1 and +2 as biological estimates based on the AMPA and GABA reversal potentials at positive and negative voltages (Rogers and Rogers, 1987); for the *fixed* versions of the models the values were kept constant whilst for the *trainable* versions these values were learnable parameters in the network, and equal inside each layer.

Next, we tested the models on the standard Spiking Heidelberg Digits (SHD) classification task. The spiking RNNs were trained offline with supervised learning for two different-sized networks: Architecture 1 (Arch-1L), which had one hidden layer (and a readout layer); and Architecture 2 (Arch-2L), which contained two hidden layers (and a readout layer). The SRNNs were trained via BPTT (see “Methods” section). We evaluated task performance on the test set through *accuracy*; and energy consumption through two measures, which describe practically useful energy measures when implementing these networks: *Scaled Median Absolute Deviation (SMAD)* and *Spike energy consumption*. These energy measures estimate the average energy consumption per trial by weighing the mean network activity, $\bar{s_{t}}$, and the mean synaptic transmission of the network caused by said spiking activity, $\bar{\tau_{t}}$. This energy measure reflects the fact that energy consumption in biological neural networks is both driven by the spike itself as well as the synaptic activity caused by the spiking (Levy and Baxter, 1996; Lennie, 2003); note that we are not concerned here with energy consumption in neuromorphic chips. In more detail, the latter energy measure can be described for a particular sample m and time point t:


$$\begin{array}{ll} E_{t}^{m} & =c_{1}\bar{s_{t}^{m}}+c_{2}\bar{\tau_{t}^{m}}\\ \bar{E_{t}} & =\overset{M}{\underset{m=1}{\sum }}c_{1}\bar{s_{t}^{m}}+c_{2}\bar{\tau_{t}^{m}}\\ E_{total} & =\underset{t}{\sum }\bar{E_{t}} \end{array}$$

where


$$\begin{array}{ll} \tau_{t}^{m} & =|{\text{s}}_{t}^{m}|⊗|\text{W}|\\ \left|\text{W}\right| & =\underset{j}{\sum }|W_{ji}| \end{array}$$

Here, *c*_1_, *c*_2_ are constants that weigh the impact of activity and synaptic transmission (following Ali et al. (2022) $c_{1}=\frac{1}{3},c_{2}=\frac{2}{3}$). We can inversely correlate this measure with network sparseness and thus with the network's energy efficiency (see “Methods” section for details).

Table 1 summarizes the accuracy and energy efficiency of the different models.

**Table 1:** Results for different models for two different size networks.

Architecture	Model	Accuracy (%)	Test spike energy	SMAD
1 Layer	Base	92.36 ± 0.76	1065.23 ± 10.68	0.5409 ± 0.0067
	Dale	90.78 ± 0.40	807.18 ± 27.96	0.4965 ± 0.0107
	Train AM+GA	92.25 ± 0.21	340.04 ± 9.32	0.3626 ± 0.0160
	Train AMPA	92.17 ± 0.46	657.99 ± 25.88	0.4395 ± 0.0193
	Train GABA	92.10 ± 0.65	470.67 ± 14.66	0.3881 ± 0.0150
	Fixed AM+GA	91.52 ± 1.21	345.05 ± 26.35	0.3259 ± 0.0127
	Fixed AMPA	92.12 ± 0.82	638.37 ± 10.08	0.4226 ± 0.0209
	Fixed GABA	92.15 ± 0.49	462.26 ± 21.35	0.3495 ± 0.0102
2 Layers	Base	89.35 ± 1.11	818.52 ± 35.00	0.4952 ± 0.0197
	Dale	90.06 ± 1.04	623.42 ± 14.45	0.4380 ± 0.0139
	Train AM+GA	91.87 ± 0.20	305.51 ± 8.65	0.3286 ± 0.0146
	Train AMPA	91.36 ± 0.86	529.63 ± 26.37	0.3893 ± 0.0211
	Train GABA	91.33 ± 0.26	459.86 ± 21.65	0.3988 ± 0.0178
	Fixed AM+GA	91.93 ± 1.09	298.52 ± 16.83	0.3302 ± 0.0039
	Fixed AMPA	90.94 ± 0.53	509.52 ± 23.93	0.3711 ± 0.0189
	Fixed GABA	90.97 ± 0.44	430.21 ± 13.58	0.3877 ± 0.0126

The number of layers on the left refers to the number of hidden layers in the trained networks. *Accuracy* and *Voltage fluctuation* relate to performance on the test set averaged across 5 iterations of the model; *Voltage fluctuation* is measured using the SMAD. The *train* (trainable) models had learnable reversal thresholds whilst the *fixed* models kept the values at our biological estimates.

In comparison to the *baseline model*, the *Dale model* showed a reduction in spike energy and voltage fluctuations for both the Arch-1L and Arch-2L. Introducing the voltage reversal potential led to a substantial and systematic reduction in both energy consumption and voltage ranges compared to the *baseline* and *Dale models*. Also, the differences in the mean value between the *fixed* and their *dynamic* counterparts were non-significant across the models (except for the voltage fluctuations in the *AM+GA* and *GABA* models).

**Figure 2:**
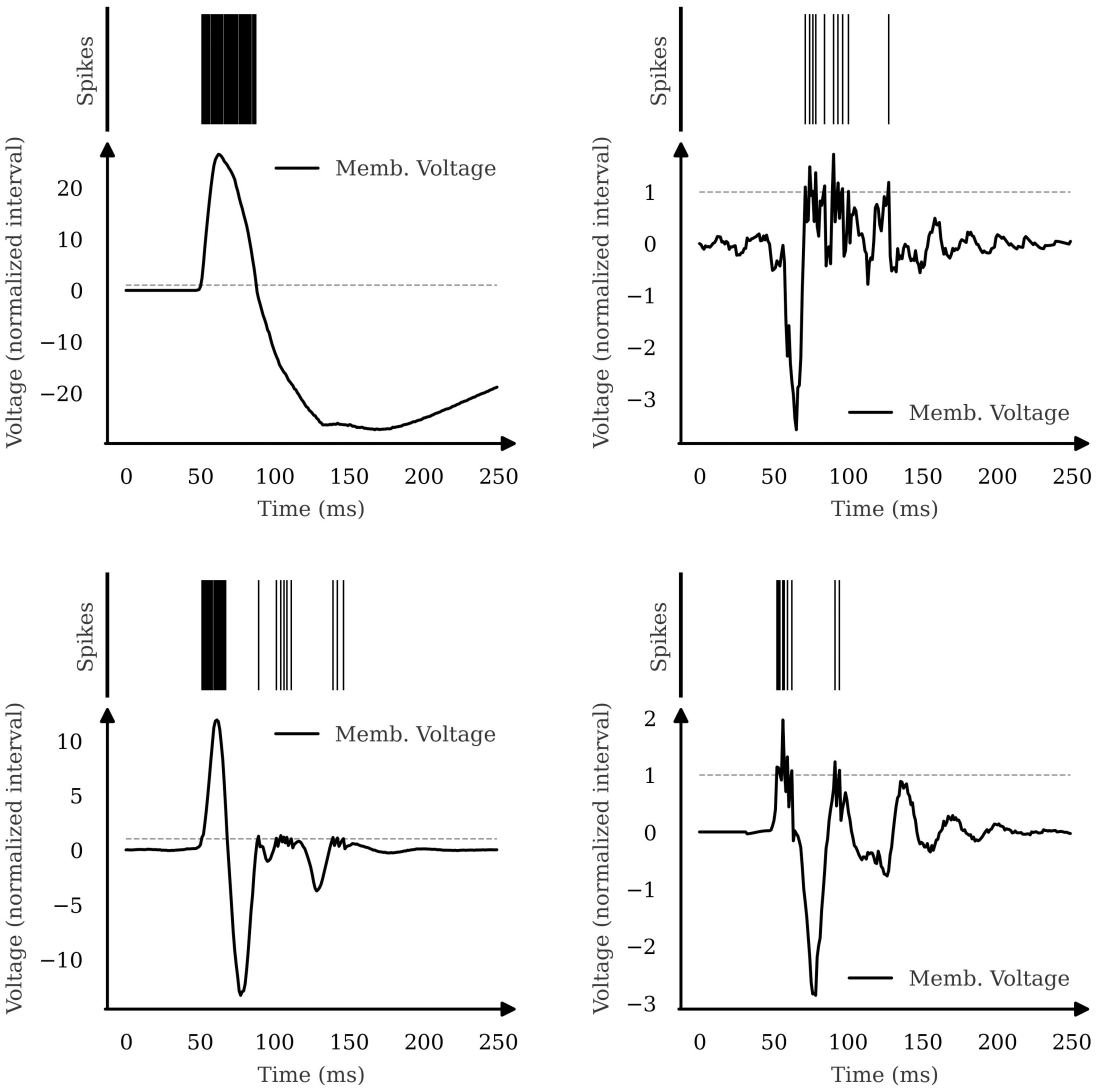
On the left panels, we have examples of non-biologically constrained models' neuron profiles for 1 trial. On the right panels, we have the same but this time for the model constrained with Dale's law and reversal potentials. The normalized interval has the resting potential at 0 and the spike threshold at 1.

In Arch-1L, we observed an approximately 3-fold reduction in spiking energy, and a 1.5-fold reduction in the *SMAD* of the membrane potential fluctuations for the *AM+GA models* as compared to the *baseline* and *Dale's model*. In Arch-2L, we observed similar improvements in energy efficiency. Thus, neurons fired more sparsely (see Tables 2, 3 and Supplementary Figures S1, S2) and had a narrower distribution of voltages in the model with reversal potentials. Analysis of the separate effects of AMPA and GABA suggests that both tend to reduce spiking energy and voltage fluctuations, but act additively when combined (Table 1). To illustrate these effects, we show some of the neuron responses (Figure 2) for the *baseline network* with large membrane potential fluctuations, compared to sparser firing and constrained membrane potentials with the voltage reversal potential for the *AM+GA model*.

**Table 2:** Parameters across neurons for different models after training.

Architecture	Parameters	Train AM+GA	Fixed AM+GA	Dale	Baseline
1 Layer	α	0.894 ± 0.001	0.891 ± 0.003	0.883 ± 0.002	0.887 ± 0.001
	β	0.992 ± 0.000	0.991 ± 0.001	0.992 ± 0.000	0.991 ± 0.000
	*a*	0.405 ± 0.017	0.422 ± 0.016	0.347 ± 0.007	0.356 ± 0.009
	*b*	0.573 ± 0.030	0.587 ± 0.033	0.542 ± 0.026	0.563 ± 0.027
	|λ|	0.839 ± 0.007	0.841 ± 0.007	0.935 ± 0.001	0.937 ± 0.001
	Frequency [Hz]	5.635 ± 0.173	5.883 ± 0.378	7.770 ± 0.190	7.720 ± 0.138
	Fraction oscillatory	0.266 ± 0.023	0.243 ± 0.070	0.845 ± 0.018	0.854 ± 0.006
	CV	1.036 ± 0.028	0.966 ± 0.010	1.359 ± 0.010	1.422 ± 0.022
	FR [Hz]	17.355 ± 0.500	16.907 ± 0.685	23.146 ± 0.744	24.495 ± 0.386
2 Layers	α	0.904 ± 0.002	0.902 ± 0.002	0.898 ± 0.003	0.901 ± 0.002
	β	0.992 ± 0.000	0.992 ± 0.000	0.992 ± 0.000	0.993 ± 0.000
	*a*	0.415 ± 0.010	0.409 ± 0.014	0.390 ± 0.015	0.390 ± 0.012
	*b*	0.743 ± 0.029	0.730 ± 0.039	0.767 ± 0.042	0.758 ± 0.023
	|λ|	0.861 ± 0.006	0.858 ± 0.010	0.944 ± 0.001	0.945 ± 0.001
	Frequency [Hz]	6.085 ± 0.120	5.990 ± 0.260	7.653 ± 0.238	7.500 ± 0.213
	Fraction oscillatory	0.423 ± 0.032	0.416 ± 0.028	0.870 ± 0.015	0.874 ± 0.009
	CV	1.232 ± 0.030	1.200 ± 0.032	1.502 ± 0.031	1.587 ± 0.044
	FR [Hz]	16.734 ± 0.556	16.365 ± 0.716	20.206 ± 1.182	22.407 ± 0.979

For two-layer architectures, values are averaged across layers. Values are mean ± std across iterations. For the train AM+GA and fixed AM+GA models, |λ|, frequency, and fraction oscillatory were computed from the conductance-dependent dynamics.

**Table 3:** Parameters for 2-layer architectures split per layer.

		Train AM+GA		Fixed AM+GA		Dale		Baseline	
Architecture	Parameters	L1	L2	L1	L2	L1	L2	L1	L2
2 Layers	α	0.906 ± 0.003	0.901 ± 0.002	0.904 ± 0.001	0.900 ± 0.003	0.898 ± 0.002	0.899 ± 0.003	0.901 ± 0.003	0.901 ± 0.002
	β	0.990 ± 0.000	0.995 ± 0.000	0.990 ± 0.001	0.994 ± 0.000	0.990 ± 0.000	0.995 ± 0.000	0.991 ± 0.001	0.994 ± 0.000
	*a*	0.384 ± 0.013	0.445 ± 0.007	0.394 ± 0.013	0.424 ± 0.016	0.396 ± 0.015	0.383 ± 0.015	0.399 ± 0.015	0.382 ± 0.010
	*b*	0.821 ± 0.023	0.666 ± 0.034	0.829 ± 0.032	0.631 ± 0.046	0.849 ± 0.030	0.685 ± 0.055	0.841 ± 0.024	0.675 ± 0.021
	|λ|	0.869 ± 0.009	0.853 ± 0.003	0.865 ± 0.013	0.851 ± 0.008	0.942 ± 0.001	0.945 ± 0.002	0.945 ± 0.001	0.946 ± 0.001
	Frequency [Hz]	5.793 ± 0.145	6.378 ± 0.095	5.905 ± 0.250	6.073 ± 0.270	7.748 ± 0.195	7.555 ± 0.280	7.495 ± 0.265	7.503 ± 0.160
	Fraction oscillatory	0.351 ± 0.041	0.496 ± 0.022	0.364 ± 0.037	0.468 ± 0.018	0.868 ± 0.016	0.872 ± 0.014	0.881 ± 0.013	0.867 ± 0.005
	CV	1.079 ± 0.024	1.385 ± 0.036	1.054 ± 0.025	1.345 ± 0.038	1.476 ± 0.030	1.528 ± 0.032	1.581 ± 0.045	1.592 ± 0.043
	FR [Hz]	17.122 ± 0.458	16.345 ± 0.653	16.333 ± 0.901	16.397 ± 0.531	17.691 ± 0.973	22.721 ± 1.391	20.005 ± 0.799	24.808 ± 1.158

Values are mean ± std across iterations. For the train AM+GA and fixed AM+GA models, |λ|, frequency and fraction oscillatory were computed from the conductance-dependent dynamics.

Next, we examined the effect on task performance.

Introducing the *Dale model* for Arch-1L significantly reduced the accuracy as compared to the baseline model, with a reduction of close to 1.5%, typical of introducing Dalean constraints on neural networks. For Arch-2L, the accuracy did not significantly change from the baseline to the Dale model.

However, the accuracy drop in the Arch-1L Dale network as compared to the base model was recovered by introducing the reversal potentials; strikingly for Arch-2L there was a significant increase in accuracy using the *AM+GA models* compared to the baseline and Dale models.

Analysis of the performance suggests that including either an *AMPA* or *GABA* reversal potential is enough to get accuracy close to the full *train AM+GA model*; however, best performance and energy reduction are obtained by including both.

Notably, even though there is no significant difference in the mean values of the *fixed/train/baseline*(for Arch-1L), the std between iterations is significantly smaller across all measures for the *train AM+GA model*, suggesting more consistent performance across model iterations.

The learned values for the reversal thresholds are aligned with our biological priors and are consistent between *AM+GA models* and the individual AMPA/GABA models (Table 4).

**Table 4:** Excitatory and inhibitory thresholds θ across architectures and model types.

Architecture	Model	θ_exc_	θ_inh_
1 Layer		2.688 ± 0.103	−1.777 ± 0.170
		2.573 ± 0.075	N/A
		N/A	−2.036 ± 0.151
2 Layers	L1	2.227 ± 0.021	−1.021 ± 0.025
	L2	2.437 ± 0.076	−1.351 ± 0.105
	L1	2.183 ± 0.027	N/A
	L2	2.470 ± 0.106	N/A
	L1	N/A	−1.334 ± 0.124
	L2	N/A	−1.222 ± 0.107

Rows with N/A in θ_inh_ correspond to AMPA-only models; rows with N/A in θ_exc_ correspond to GABA-only models; rows with both values correspond to AM+GA models.

### 2.1 Analysis of unit properties

To understand how reversal potentials affect neuronal activity in the network, we conducted further theoretical analyses of adapLIF neurons with reversal potentials. As previously shown (Baronig et al., 2025), adapLIF neurons can exhibit resonances at different frequencies, a result of their being a system of two coupled differential equations that can exhibit subthreshold resonance. The performance gain observed with adapLIF neurons likely relates to increased heterogeneity and time constants (Bellec et al., 2018). However, subthreshold resonance may also play a role because RNNs with units configured as damped harmonic oscillators coupled oscillator RNN (CoRNNs) have potential theoretical benefits and enhance performance on various tasks (Rusch and Mishra, 2021), in particular when units show resonance at the frequency of the external input (Effenberger et al., 2025).

By analyzing neural properties (see “Methods” section and Supplementary Figures S1, S2), we found that neurons exhibited a broad distribution of oscillatory frequencies in the range 0–16 Hz (see Supplementary Figures S3, S4), suggesting that the network learns substantially heterogeneous resonance responses. The *AM+GA models* with reversal potentials had substantially fewer oscillatory units than the *Dale's model* and the *baseline model*, with lower resonance frequencies (Table 2). Thus, the improved energy efficiency and performance were not associated with increased oscillatory activity but rather with greater heterogeneity between oscillatory and non-oscillatory activity.

Furthermore, in Arch-2L, the *AM+GA models* also had spike patterns closer to Poisson processes (CV closer to 1, Tables 2, 3) which translates to spike train statistics that match biological neurons (Dayan and Abbott, 2005). Notably, the AM+GA model exhibited more adaptation in response to subthreshold activity, with larger values of *a*.

It can be shown that the inclusion of a reversal potential makes the neuron's properties dynamic and dependent on the time-varying input, rather than static. To see this, we note that our system of equations is written as


$$\begin{array}{l} h_{t}=\alpha h_{t-1}+\left(1-\alpha \right)\left(-w_{t-1}+I_{t}\right)-V_{threshold}S_{t-1}\\ w_{t}=\beta w_{t-1}+ah_{t}+bS_{t}\\ I_{j}^{t}=\overset{n}{\underset{i}{\sum }}W_{ji}^{in}x_{i}^{t}+\overset{n_{exc}}{\underset{i\neq j}{\sum }}W_{ji}\left(\theta_{exc}-h_{j}^{t-1}\right)S_{i}^{t-1}\\ \;+\overset{n_{inh}}{\underset{i=n_{exc}+1\neq j}{\sum }}W_{ji}\left(h_{j}^{t-1}-\theta_{inh}\right)S_{i}^{t-1} \end{array}$$

If we consider only one neuron *j* and it is under subthreshold dynamics, we can simplify these equations into:


$$\begin{array}{l} h_{t}=\alpha h_{t-1}+\left(1-\alpha \right)\left(-w_{t-1}+I_{t}\right)\\ w_{t}=\beta w_{t-1}+ah_{t}+bS_{t}\\ I_{t}=I_{ind}-J\left({\text{S}}_{t-1},W\right)h_{t-1} \end{array}$$

Here, $I_{ind}=\overset{n}{\underset{i}{\sum }}W_{ji}^{in}x_{i}^{t}+\overset{n_{exc}}{\underset{i\neq j}{\sum }}W_{ji}\theta_{exc}S_{i}^{t-1}-\overset{n_{inh}}{\underset{i=n_{exc}+1\neq j}{\sum }}W_{ji}\theta_{inh}S_{i}^{t-1}$ has the feedforward component and the recurrent component independent of *h*_*j*_ and $J\left({\text{S}}_{t-1},W\right)=\overset{n_{exc}}{\underset{i\neq j}{\sum }}W_{ji}S_{i}^{t-1}-\overset{n_{inh}}{\underset{i=n_{exc}+1\neq j}{\sum }}W_{ji}S_{i}^{t-1}$ represents the recurrent gain dependent on the membrane potential. Note that *J* > 0 and that *J* represents the sum of the magnitudes of excitatory and inhibitory conductances. We can then write the state transition matrix by plugging in *h*_*t*_→*w*_*t*_:

Here, we can see that the inputs themselves determine the state transition matrix *A*, making the response property of the neuron input-dependent, where


$$\begin{array}{l} A=\left[\begin{array}{c} \alpha -\left(1-\alpha \right)J\;-\left(1-\alpha \right)\\ a\left(\alpha -\left(1-\alpha \right)J\right)\;\beta -a\left(1-\alpha \right) \end{array}\right] \end{array}$$

To understand the impact of *J* (the amount of conductance), we perform an eigenvalue analysis in the subtreshold regime (see the “Methods” section for exact derivations). This analysis shows oscillation magnitudes and frequencies that are dependent on recurrent network activity itself, as it determines *J* as a function of time for each neuron.

The expression for the eigenvalue magnitudes equals (see “Methods” section)


$$\begin{array}{l} |\lambda |=\sqrt{\det A}=\sqrt{\beta \left[\alpha -\bar{\alpha }J\right]} \end{array}$$ (1)

Thus, increased conductance *J* strictly decreases the magnitude of subthreshold oscillations, because β>0 and $\bar{\alpha }>0$. This reduction in amplitude is related to the classic shunting effect of synaptic conductance in biophysics (Koch, 2004).

The eigenvalues are complex as long as


$$\begin{array}{l} \Delta_{A}=\mathrm{Tr}{\left(A\right)}^{2}-4\det\left(A\right)<0 \end{array}$$ (2)

The discriminant is an upward parabola as a function of *J*, reaching a minimum at J^*^; that is, the system is most likely to oscillate at this point. Oscillatory behavior requires *J* to lie in the interval {*J*_−_, *J*_+_}, where


$$\begin{array}{l} J_{\pm }=J^{*}\pm 2\sqrt{\beta \frac{a}{\bar{\alpha }}} \end{array}$$ (3)

and


$$\begin{array}{l} J^{*}=\frac{\alpha -\beta -a\bar{\alpha }}{\bar{\alpha }}. \end{array}$$ (4)

We find that all of the neurons in the trained model have J^*^ < 0.

Hence, increased conductance *J* invariantly decreases the oscillation magnitude; the eigenvalue magnitudes keep decreasing until the system is no longer oscillatory.

Thus, the best performing model *train AM+GA*, which has more oscillatory units for *J* = 0 (see Supplementary Tables S4, S5), shows that the tendency to oscillate decreases dramatically with increased *J*. Note that it is required that *a* ≥ 0 for the system to exhibit oscillations (see “Methods” section). As *a* → 0, the window of conductances *J* in which oscillations occur becomes increasingly narrow.

Finally, for complex discrete eigenvalues we have a frequency $f=\frac{\omega }{2\pi }f_{s}$ with $\omega_{c}=\arccos\left(\frac{\frac{1}{2}\text{Tr(}A)}{\sqrt{\det A}}\right)$ (note that deriving the frequency from continuous dynamics as done in other study is inaccurate). We obtain that the maximum frequency is obtained for


$$\begin{array}{l} J_{c}=\frac{\alpha +\bar{\alpha }a-\beta }{\bar{\alpha }}. \end{array}$$

As a consequence, the frequencies may initially increase slightly as a function of *J* up to *J*_*c*_, then decrease until the system stops oscillating at *J*^+^, at which point the frequencies are zero. For our range of parameters, on average *J*_*c*_ ≤ 0 (see Table 2), such that frequencies will on average decrease with increased conductance *J*. In fact, for *AM+GA model* we compute the mean neuron parameters with our dynamic values of *J* and averaged them for Arch-1L and Arch-2L, in Tables 2, 3, which yield significantly lower results when compared to the static (no *J* taken into consideration) results (see Supplementary Table S4).

In sum, the *AM+GA models* with reversal potentials have substantially fewer oscillatory units with weaker oscillations. The reversal potential further leads to weaker oscillations and a non-linear dependence of frequency on the conductance *J*.

## 3 Discussion

The performance of SRNNs has been improved due to the introduction of several features, including the use of surrogate gradients to implement back-propagation-through-time (BPTT) (Werbos, 2002); units showing adaptation mechanisms (Bellec et al., 2018); increased heterogeneity of single-neuron properties (Perez-Nieves et al., 2021; Bittar and Garner, 2022). However, many properties of biological neurons have yet to be implemented in SRNNs, and the effects of these properties on their performance and energy efficiency remain partially unexplored. The implementation of this adapLIF in a task-solving RSNN (Bittar and Garner, 2022; Baronig et al., 2025) still results in non-biologically plausible voltage dynamics, as subthreshold values fluctuate outside the biological range. In biological neurons, voltage dynamics are constrained by specific mechanisms, namely the existence of reversal potentials (Koch, 2004). Here, we define a new neuron model in which GABAergic and glutamatergic neurons give rise to recurrent synaptic inputs that are governed by a reversal potential. The SRNN thus contains two classes of neurons that mimic Dale's law (i.e., neurons that use a single neurotransmitter), which are inhibitory or excitatory depending on the receiving neuron's membrane potential.

We trained SRNNs on the Spiking Heidelberg Digits and show that SRNNs with reversal potentials cut spike energy by up to 3 × , while maintaining or increasing task accuracy. We provide a theoretical analysis of the model's dynamics and examine the subthreshold resonance of adapLIF neurons. Improved model performance with reversal potential is concomitant with a reduction in the number of oscillatory units and a decrease in oscillation frequency compared with the baseline model. The reversal potential makes subthreshold resonance conductance dependent, such that increased conductance reduces oscillatory behavior and decreases frequency.

Further study is needed to precisely identify the reasons why including the reversal potential increases task performance and energy efficiency. There may be several reasons: (1) The reversal potential naturally constrains the voltage range of the neurons, which is explained by the fact that the sign of excitation and inhibition changes around the reversal potential, thereby improving the energy efficiency. This may improve task performance by keeping voltages within the range where the surrogate gradients do not vanish, thereby mitigating the vanishing-gradient problem. In agreement, a previous study suggests that clipping a neuron's voltage distribution can improve the performance of a feedforward SNN (Guo et al., 2022). (2) Mathematically, the reversal potential allows, for example, inhibitory input to have divisive effects on excitatory inputs rather than just subtractive effects, thus enhancing the computational capacity of the network (Koch, 2004). That is, the reversal potential adds a nonlinear integration of inputs inside the neuron, which may enhance the computational capacity of neurons. (3) We showed that the reversal potential allows for the resonance frequencies of the individual neurons to become input-dependent. Hence, a neuron's resonance function may change in response to input, thereby creating more complex, time-dependent feature-selective responses tuned to specific input patterns.

Reversal potentials are, biologically, an unavoidable consequence of the electrochemical gradient of ion channels defined by intracellular and extracellular concentrations. For example, GABA is inhibitory because the intracellular chloride concentration is much lower than the outside of the cell, such that the chemical force is stronger than the electric force when the voltage is above the reversal potential (Koch, 2004). In fact, this is a property that depends on the KCC2 chloride receptor and changes through development, but can also vary between areas (e.g., dentate gyrus) (Ben-Ari, 2007; Sauer et al., 2012). Thus, it stands to reason that the precise GABAergic reversal is a property that is evolutionarily optimized. In this study, we showed both a fixed biological estimate approach, as well as optimal value convergence, which brought more consistent results across different weight initializations and can be an indicator that biological tuning around these thresholds brings consistent energy efficiency/performance across individual areas.

In this study, we report an increase in performance by incorporating Dale's law with reversal potentials; however, there are other methods that may improve task performance that we did not examine here. Baronig et al. (2025) includes a biologically unrealistic *q* parameter in the adaptation equation (see “Methods” section) that needs to be adjusted for each architecture. They also perform additional preprocessing on the inputs, strongly reducing the number of input channels.

Recently, there has been some interest in the ability of RNNs with oscillatory units (coupled oscillator RNN) to improve task performance (Rusch and Mishra, 2021; Keller and Welling, 2023; Effenberger et al., 2025; Baronig et al., 2025; Lanthaler et al., 2023). The subthreshold dynamics of adLIF neurons can produce oscillatory dynamics (Baronig et al., 2025), with subthreshold resonance around relatively low frequencies around 5–8 Hz (i.e., in the delta range). (Note that we improved the frequency calculation of these units, taking into account the discrete nature of the dynamics.) In general, it is unclear whether the tendency to oscillate or rather the introduction of an additional current/self-influence accounts for possible increases in performance in RNNs with oscillatory units compared to RNNs without oscillatory units (Rusch et al., 2021); noting that a damped harmonic oscillator can be broken up in two first order equations, one with a fast and one with a slow component that allows learning long temporal dependencies. Interestingly, we showed that reversal potentials strongly reduced the fraction of oscillatory units (e.g., from 0.85 to 0.26 in a one-layer neural network) and the magnitudes of the eigenvalues, even though task performance and energy efficiency improved compared to the Dale network. The fact that better performance was obtained with far fewer oscillatory units but similar time constants suggests that the propensity to oscillate may not, *per se*, boost task performance or energy efficiency. In the networks trained here, the propensity to oscillate was strongly reduced by the input stimulus, leading to a marked increase in conductance. Interestingly, this shows some similarity to biological neurons and networks of neurons, which tend to show more low-frequency activity in the absence of sensory stimulation (i.e., during rest), while stimulus-evoked activity classically shows desynchronization of neural responses (Buzsáki, 2006).

In sum, the findings here are another step toward greater biological realism in spiking neural networks, enabling efficient, high-performance training with Dale's law. These findings likely have important applications for neuromorphic computing technologies, in which energy efficiency and network sparseness play an important role.

## 4 Methods

### 4.1 Neuron model

We ran a discretized version of the adapLIF model, using the symplectic-Euler (SE) discretization (Baronig et al., 2025). Some small changes to the dynamics were made compared to those in the studies by Bittar and Garner (2022); Baronig et al. (2025), as we will discuss below. The discrete update equations according to the SE-discretization method are:


$$\begin{array}{l} {ĥ}_{t}=\alpha h_{t-1}+\left(1-\alpha \right)\left(-w_{t-1}+I_{t}\right) \end{array}$$ (5)

$$\begin{array}{l} S_{t}=H\left({ĥ}_{t}-V_{threshold}\right) \end{array}$$ (6)

$$\begin{array}{l} h_{t}={ĥ}_{t}\left(1-S_{t}\right) \end{array}$$ (7)

$$\begin{array}{l} w_{t}=\beta w_{t-1}+\left(1-\beta \right)\left(ah_{t}+bS_{t}\right) \end{array}$$ (8)

with *h* the hidden membrane potential, *S*_*t*_ a binary variable for the spike output of the neuron, *w* the adaptation variable, and *I* the input currents to the neuron at that time step. The neuron parameters α, β, *a*, and *b* correspond to the membrane decay rate, adaptation decay rate, and sub- and supra-threshold gain constants, respectively; these parameters remain fixed post-learning. We initialize them over a biologically plausible range based on studies by Bittar and Garner (2022); Baronig et al. (2025) and allow them to train individually per neuron to add heterogeneity to the network (Perez-Nieves et al., 2021).

The discrete update equations we used for the simulations are:


$$\begin{array}{l} h_{t}=\alpha h_{t-1}+\left(1-\alpha \right)\left(-w_{t-1}+I_{t}\right)-V_{threshold}S_{t-1} \end{array}$$ (9)

$$\begin{array}{l} S_{t}=H\left(h_{t}-V_{threshold}\right) \end{array}$$ (10)

$$\begin{array}{l} w_{t}=\beta w_{t-1}+ah_{t}+bS_{t} \end{array}$$ (11)

#### 4.1.1 Differences from previous works

There are two major differences from Equations 5–11. First, we avoid the hard reset, Equation 7 used in the study by Baronig et al. (2025), as this represented in preliminary tests a 7% drop off in accuracy, and use instead a soft reset similar to the one used in the study by Bittar and Garner (2022), by adding “−*V*_*threshold*_***S***_*t*−1_” in Equation 9.

Second, we drop the (1−β) term in Equation 8 as in preliminary tests the network did not learn efficiently: accuracy peaked [55%, 60%] [compared to (85%, 95%) we get otherwise]. This is due to (1−β) being very small (in our range of values), effectively removing the dynamical component of our adaptation variable. The scaling of (1−β) was solved in the study by Bittar and Garner (2022) identically to us (seen in the source code, as in the original paper they have that scaling factor present); and in the study by Baronig et al. (2025) by adding another scaling factor *q*, reshaping Equation 8 to:


$$\begin{array}{l} w_{t}=\beta w_{t-1}+q\left(1-\beta \right)\left(ah_{t}+bS_{t}\right) \end{array}$$ (12)

We chose to remove the term from our main results since Baronig et al. (2025) suggests *q* needs to be tuned for each task as a global gain on the neuron's adaptation constants. In fact, removing this factor can be seen as absorbing the (1−β) factor into the arbitrary parameters *a* and *b*, which rescales the values of the initialization intervals for *a* and *b*, and whose bounds we have discussed previously, and in more detail below.

#### 4.1.2 Additions to the neuron model

To the baseline equations, Equations 9–11, we added two biologically realistic features: (1) Dale's law; and (2) a voltage reversal potential.

(1) *Dale's law* restricts the communication of a neuron to a single neurotransmitter for all postsynaptic connections. This means, for our computation, that we divide our nodes into two subpopulations, with their recurrent weights either positive (excitatory neurons) or negative (inhibitory neurons).

The two populations comprise 50% of the population in this case, implemented to introduce an inductive bias toward balanced E/I.

(2) By using a *reversal potential*, dendritic inputs to neurons are scaled by their local voltage potential. Its existence is well established in the neuroscientific literature (Brown et al., 1971; Mofidi et al., 2020; Eisenberg et al., 2015; Koch, 2004).

To model the reversal potential, we changed our input current equation to:


$$\begin{array}{l} I_{j}^{t}=\overset{n}{\underset{i}{\sum }}W_{ji}^{in}x_{i}^{t}\\ +\overset{n_{exc}}{\underset{i\neq j}{\sum }}W_{ji}^{recurrent}\left(\theta_{exc}-h_{j}\right)S_{i}^{t-1}\\ +\overset{n_{inh}}{\underset{i=n_{exc}+1\neq j}{\sum }}W_{ji}^{recurrent}\left(h_{j}-\theta_{inh}\right)S_{i}^{t-1} \end{array}$$

### 4.2 Theoretical derivations of the neuron model

The system of equations in the subthreshold regime is given by


$$\begin{array}{l} h_{t}=\alpha h_{t-1}+\bar{\alpha }\left(-w_{t-1}+I_{t}\right)\\ w_{t}=\beta w_{t-1}+ah_{t}+bS_{t}\\ I_{t}=I_{ind}-J\left({\text{S}}_{t-1},W\right)h_{t-1} \end{array}$$

where $\bar{\alpha }=\left(1-\alpha \right)$. Here, $I_{ind}=\overset{n}{\underset{i}{\sum }}W_{ji}^{in}x_{i}^{t}+\overset{n_{exc}}{\underset{i\neq j}{\sum }}W_{ji}\theta_{exc}S_{i}^{t-1}-\overset{n_{inh}}{\underset{i=n_{exc}+1\neq j}{\sum }}W_{ji}\theta_{inh}S_{i}^{t-1}$, is the feedforward component plus the recurrent component independent of *h*_*j*_.

$J\left({\text{S}}_{t-1},W\right)=\overset{n_{exc}}{\underset{i\neq j}{\sum }}W_{ji}S_{i}^{t-1}-\overset{n_{inh}}{\underset{i=n_{exc}+1\neq j}{\sum }}W_{ji}S_{i}^{t-1}$, is the recurrent gain dependent on the membrane potential.

Setting *S*_*t*_ = 0 (subthreshold regime), the system of equations can be written as:


$$\begin{array}{l} h_{t}=\left(\alpha -\bar{\alpha }J\right)h_{t-1}-\bar{\alpha }w_{t-1}+\bar{\alpha }I_{ind}\\ w_{t}=a\left(\alpha -\bar{\alpha }J\right)h_{t-1}+\left(\beta -a\bar{\alpha }\right)w_{t-1}+a\bar{\alpha }I_{ind} \end{array}$$

We can then define the subtreshold state transition matrix *A*,


$$\begin{array}{l} A=\left[\begin{array}{c} \alpha -\bar{\alpha }J-\bar{\alpha }\\ a\left(\alpha -\bar{\alpha }J\right)\beta -a\bar{\alpha } \end{array}\right] \end{array}$$

We have


$$\begin{array}{l} \lambda =\frac{1}{2}\mathrm{Tr}\left(A\right)\pm \frac{1}{2}\sqrt{\mathrm{Tr}{\left(A\right)}^{2}-4\det\left(A\right)} \end{array}$$ (13)

and


$$\begin{array}{l} \mathrm{Tr}\left(A\right)=\alpha +\beta -\bar{\alpha }\left(a+J\right) \end{array}$$ (14)

$$\begin{array}{l} \det\left(A\right)=\beta \left[\alpha -\bar{\alpha }J\right] \end{array}$$ (15)

#### 4.2.1 Eigenvalue magnitude

We first consider the case that there is a pair of complex eigenvalues, that is, the neuron exhibits subthreshold resonance (which occurs in more than half of the measured neurons). In this case, we have the eigenvalue magnitude


$$\begin{array}{l} |\lambda |=\sqrt{\det A}=\sqrt{\beta \left[\alpha -\bar{\alpha }J\right]} \end{array}$$ (16)

Hence, increased recurrent input conductance *J* tends to decrease the magnitude of subthreshold oscillations as β > 0 and $\bar{\alpha }>0$.

#### 4.2.2 Effect of input on tendency to oscillate

The eigenvalues are complex as long as


$$\begin{array}{l} \Delta_{A}=\mathrm{Tr}{\left(A\right)}^{2}-4\det\left(A\right)<0 \end{array}$$ (17)

The discriminant is an upward parabola, reaching a minimum at J^*^, that is, the system is most likely to oscillate at this point, with complex eigenvalues in the interval


$$\begin{array}{l} J_{\pm }=J^{*}\pm 2\sqrt{\beta \frac{a}{\bar{\alpha }}} \end{array}$$ (18)

where


$$\begin{array}{l} J^{*}=\frac{\alpha -\beta -a\bar{\alpha }}{\bar{\alpha }}. \end{array}$$ (19)

We can see that J^*^ < 0 typically over our range of parameters (Table 4). As the conductance *J* increases, there is a conductance value *J*^+^ at which point the neuron stops oscillating. Hence, an increase in *J* leads to a decrease in eigenvalue magnitudes until the neuron falls outside the range of oscillatory behavior.

Increased adaptation *a* widens the window in which oscillations occur; increased membrane potential time constant (α) also increases the window. If *a* > 0, there exists an interval *J* ∈ (*J*_−_, *J*_+_) in which the eigenvalues are complex, since $\Delta_{A}\left(J\right)={\bar{\alpha }}^{2}{\left(J-J^{*}\right)}^{2}-4\beta a\bar{\alpha }$. In the absence of an input, *J* = 0, we can see that Δ_*A*_ < 0 if


$$\begin{array}{l} a_{-}<a<a_{+},\;a_{-},a_{+}>0 \end{array}$$ (20)

Here


$$\begin{array}{l} a_{\pm }=\frac{\alpha +\beta \pm 2\sqrt{\alpha \beta }}{\bar{\alpha }}. \end{array}$$ (21)

#### 4.2.3 Bounds on the stability of the system

Stability is given by the Schur inequalities.


$$\begin{array}{l} 1-\mathrm{Tr}A+\det A>0\Rightarrow a\bar{\alpha }>-\left[\bar{\alpha }-\beta +\alpha \beta +\bar{\alpha }\left(1-\beta \right)J\right]. \end{array}$$ (22)

$$\begin{array}{l} a_{\min}\left(J\right)=-\frac{1-\alpha -\beta +\alpha \beta }{1-\alpha }-\left(1-\beta \right)J=-\left(1-\beta \right)\left(1+J\right) \end{array}$$ (23)

If *a* > 0 and *J* > 0 then *a* > *a*_min_(*J*). This motivates the bound *a* > 0 to ensure stability.

We have from |det*A*| < 1


$$\begin{array}{l} \frac{\beta \alpha -1}{\beta \bar{\alpha }}<J<\frac{\beta \alpha +1}{\beta \bar{\alpha }}. \end{array}$$ (24)

Finally we have


$$\begin{array}{l} a_{\max}\left(J\right)=\frac{1+\alpha +\beta +\alpha \beta }{\bar{\alpha }}-\left(1+\beta \right)J,\;a<a_{\max}\left(J\right). \end{array}$$ (25)

For our ranges of values, this implies that *J*_*max*_ is very large (>10) and thus the system, given our parameters, is stable. It is further evident that as the network grows larger in size, the network weights should be scaled as $\frac{1}{N}$ to prevent *J* from growing too large.

##### 4.2.3.1 *J*'s critical point

For complex discrete eigenvalues, we have a frequency $f=\frac{\omega }{2\pi }f_{s}$. We will derive the dependency of ω on *J*. We shall use the correct expression for discrete-time dynamics (note that the continuous-time expression would not necessarily be accurate). We define the numerator/denominator of $\omega_{c}=\arccos\left(\frac{\frac{1}{2}\text{Tr(}A)}{\sqrt{\det A}}\right)$ as *g, p*, and the full argument of the arccos as *f*. We are going to be searching for the critical point with respect to *J*.


$$\begin{array}{l} \partial_{J}\omega_{c}=\partial_{J}\arccos\left(\underset{f}{\underset{︸}{\frac{g}{p}}}\right)=-\frac{\partial_{J}f}{\sqrt{1-f^{2}}} \end{array}$$

Expanding the terms and simplifying, we obtain:


$$\begin{array}{l} \partial_{J}\omega_{c}=\frac{d}{dJ}\arccos(f\left(J\right))=-\frac{f^{\prime }\left(J\right)}{\sqrt{1-f{\left(J\right)}^{2}}} \end{array}$$

Hence, the critical point occurs when *f*′(*J*) = 0. We have


$$\begin{array}{l} f^{\prime }\left(J\right)=-\frac{\bar{\alpha }\beta (\alpha -\beta +\bar{\alpha }\left(a-J\right))}{4(\beta \left(\alpha -\bar{\alpha }J\right){)}^{3/2}}\\ =-\frac{\bar{\alpha }\beta (\alpha -\beta +\bar{\alpha }\left(a-J\right))}{4p^{3}} \end{array}$$

Thus, the critical point occurs when $J_{c}=\frac{\alpha +\bar{\alpha }a-\beta }{\bar{\alpha }}$.

For the second derivative evaluated at the critical point, we have


$$\begin{array}{l} \partial_{J}^{2}\omega_{c}{|}_{J_{c}}=-\frac{f^{\prime \prime }\left(J_{c}\right)}{\sqrt{1-f{\left(J_{c}\right)}^{2}}} \end{array}$$

Note that


$$\begin{array}{l} f^{\prime \prime }\left(J_{c}\right)=\frac{{\bar{\alpha }}^{2}}{4\sqrt{\beta }{\left(\beta -\bar{\alpha }a\right)}^{3/2}}=\frac{{\bar{\alpha }}^{2}\beta }{4p{\left(J_{c}\right)}^{3}},\;p\left(J_{c}\right)=\sqrt{\beta \left(\beta -\bar{\alpha }a\right)} \end{array}$$

Thus, $f^{\prime \prime }\left(J_{c}\right)>0$, and the critical point *J*_*c*_ is always a maximum.

Note that we require that $J_{c}\leq J^{+}$, that is, we require that it lies in the domain of complex eigenvalues. Thus, if *J*_*c*_ > 0, initially frequencies increase as a function of *J*, until *J* = *J*_*c*_, after which the frequency decreases; if *J*_*c*_ < 0, frequencies always decrease. At *J*^+^, we have Tr(*A*)^2^ = 4det(*A*) and hence ω_*c*_ = 0. Hence, frequencies decrease until ω_*c*_ = 0 after which subthreshold oscillations disappear.

### 4.3 Data

We trained networks on the Spiking Heidelberg Digits (Cramer et al., 2022). This dataset consists of close to 10,000 aligned audio recordings of spoken digits (0–9) in English and German from 12 speakers, with two exclusive ones to the test set. These recordings are labeled according to *digit* and *language*, yielding a total of 20 classes. We generate the neural network inputs by binning the spike times into 4 ms intervals going from 0 until 1 s, generating a 250 step long time series. There are 700 input channels, the values of which are integers representing the number of times that a neuron fired in that time bin.

### 4.4 Task and neural network architectures

We examined the performance of different neural networks in learning to classify the SHD data in a supervised setting. We used a connected internal feedback spiking recurrent neural network (SRNN) with a final readout layer, similar to the one discussed in Bittar and Garner (2022). We used two different architectures, which had the same type of readout layer:

#### 4.4.1 Architecture 1L

Here we used a two-layer deep network (1 hidden layer + readout layer) with 256 neurons in the hidden layer, with a total number on the order of 250k trainable parameters. The input currents for each neuron $I_{j}^{t}$ are going to be of the form:


$$\begin{array}{l} I_{j}^{t}=\overset{n}{\underset{i}{\sum }}W_{ji}^{in}x_{i}^{t}+\overset{n}{\underset{i\neq j}{\sum }}W_{ji}^{recurrent}S_{i}^{t-1} \end{array}$$ (26)

with $x_{i}^{t}$ the value for the *i*th input channel. In vector notation, we have


$$\begin{array}{l} I_{t}=W_{in}x_{t}+W_{recurrent}S_{t-1} \end{array}$$ (27)

where *W*_*in*_ is the 700x256 input weight matrix and *W*_*recurrent*_ is the 256x256 recurrent weight matrix with diagonal filled with 0s.

#### 4.4.2 Architecture 2L

Here, we trained a three-layer deep network (two hidden layers + one readout layer) with 360 neurons in each hidden layer. The total number of trainable parameters on the order of 650k parameters.

We can again write the input current for each neuron $I_{t}^{l}$ where *l* refers to the hidden layer of the network:


$$\begin{array}{l} I_{t}^{l}=W_{in}^{l}x_{t}^{l}+W_{recurrent}^{l}S_{t-1}^{l} \end{array}$$ (28)

with $dim\left(W_{in}^{2}\right)=128$x128 and $x_{t}^{2}=S_{t}^{1}$.

#### 4.4.3 Readout layer and loss function

The readout layer of our network consisted of a leaky integrator neuron (LI), achieved by setting *a* = *b* = 0 and removing the reset mechanism. From this output, we would compute the loss function defined in the study by Bittar and Garner (2022). For each time step, we apply a softmax function to the hidden potentials of the readout layer across neurons, then sum these normalized values across time steps for all neurons and compute a cross-entropy loss with the target class for each trial. For each batch, *n*, the loss was computed as:


$$\begin{array}{l} o_{c}=\overset{T}{\underset{t=0}{\sum }}\text{softmax}\left(h_{c}^{t}\right)\\ L_{n}=-\overset{19}{\underset{c=0}{\sum }}\log\frac{\exp\left(o_{c}\right)}{\sum_{i=0}^{19}\exp\left(o_{i}\right)} \end{array}$$

For the reported results, we used the same regularization loss function as discussed in Bittar and Garner (2022). Additionally, for Arch-1L, we obtained similar performance using the regularization function discussed in (Zenke and Vogels, 2021) and computing $o_{c}=\max\left(\overset{t}{\underset{c}{h}}\right)$. The settings used for the Readout layer are compiled in Table 6.

#### 4.4.4 Parameter initialization

We briefly explored the hyperparameter (α, β, *a* and *b*) space but since this space is quite large and we are focused on comparability, we decided to base it on previous work with similar models (Bittar and Garner, 2022; Baronig et al., 2025). The hyperparameters were initialized uniformly across the accepted range and, during learning, were clamped to their bounds to ensure realistic dynamics.

In the hidden layers, the feedforward weights were initialized uniformly across $|w|<\frac{1}{\sqrt{fan_{in}}}$, and the recurrent weights were initialized using PyTorch's orthogonal method discussed in the study by (Saxe et al., 2013); as in state-of-the-art SRNN models (Baronig et al., 2025). For the readout layer, the weights were initialized from a normal distribution with a specified mean and standard deviation (SD).

When using Dale's law, we additionally split the recurrence matrix in half for the excitatory/inhibitory subpopulations. For the excitatory afferent weights, any negative initialized weights were swapped to their symmetric; for the inhibitory weights, the symmetric happened. When clamping was applied to this model, it was set to the minimum absolute value of the initialized weights.

#### 4.4.5 Training details

The SHD does not have a predefined validation set, so we randomly split the training set into a training and validation set (80/20%), and all our results came from the test set.

We trained all the models for 200 epochs on the SHD training set. We trained each model for 5 iterations using the same initialization. Every setting range used was fixed for each architecture, and shared between architectures (Table 5).

**Table 5:** Hidden layer settings for both architectures.

Setting name	Values
Voltage threshold	1.0
τ_*m*_ range	[5, 25]
τ_*s*_ range	[30, 300]
a range	[0.0, 1.0]
b range	[0.0, 2.0]
θ_*inh*_	−1.0
θ_*exc*_	2.0
Dropout probability	0.15
Init *W*_*i*→*h*_ with linear	True
Init *W*_*h*→*h*_ with orthogonal	True
Use adaptable parameters	True

**Table 6:** Readout layer settings.

Setting name	Readout layer
Title	garnerpaper_params_readout
Voltage_threshold	1.0
τ_*m*_	15ms
Use adaptable parameters	True
Use spike reset	False
Init *W*_*i*→*h*_ mean	N(0,0.03)
dt	1ms
a	0
b	0

Similar to the study by Baronig et al. (2025), we define $\alpha =e^{\frac{-\Delta t}{\tau_{m}}}$, and the membrane time constant as:


$$\tau_{m}=\tau_{m}^{min}+\omega_{m}\left(\tau_{m}^{max}-\tau_{m}^{min}\right)$$

The trainable parameter is ω_*m*_ ∈ [0, 1]. This is done so that all parameters are on the same order of magnitude for the ADAM optimizer. The analogous is done for the synaptic rate (β) and time constant (τ_*s*_).

The different settings only came from the number of neurons, already specified above.

The setting names are slightly different from those used in the dictionary available in the code for readability.

For the surrogate gradient we used the SLAYER function with α = 5 and *c* = 0.4 for all layers (Shrestha and Orchard, 2018).

### 4.5 Analyses

For all analyses, we computed these values for each iteration of *model X* and then computed the mean and standard deviation (SD) across the five iterations. Per iteration, we ran through the full test set via our sampling function (in batches), computing the value and saving it into an array; we would perform this loop 10 times and then average the result as our final per-iteration value.

For *Accuracy*, we took $y^{*}=\arg\underset{c}{\max}\underset{t}{\sum }\text{softmax}{\left(h_{t}\right)}_{c}$ (c denotes the classification neuron that goes from 0 to 19, and y^*^ is our guess for the label) and compared it to the label, *y*, of the class for each batch.

For *Test Spike Energy*, we computed the average neuron energy consumption at each time point and summed them for the duration of the stimulus. Our measure was inspired by the study by Ali et al. (2022), Equations 7, 8. We defined it for a particular sample m and time point t in a batch:


$$\begin{array}{l} E_{t}^{m}=c_{1}\bar{s_{t}^{m}}+c_{2}\bar{\tau_{t}^{m}} \end{array}$$ (29)

$$\begin{array}{l} \bar{E_{t}}=\overset{M}{\underset{m=1}{\sum }}c_{1}\bar{s_{t}^{m}}+c_{2}\bar{\tau_{t}^{m}} \end{array}$$ (30)

$$\begin{array}{l} E_{total}=\underset{t}{\sum }\bar{E_{t}} \end{array}$$ (31)

where


$$\begin{array}{l} \tau_{t}^{m}=|{\text{s}}_{t}^{m}|⊗|\text{W}| \end{array}$$ (32)

$$\begin{array}{l} \left|\text{W}|=\underset{j}{\sum }|W_{ji}\right| \end{array}$$ (33)

The $\bar{operator}$ is the average across neurons for a batch until $\bar{E_{t}}$. From this point on, it also includes the average across batches. Indeed, one can take the average across batches or neurons interchangeably, which is how we do it in practice. We chose this notation to maintain consistency with the original notation discussed in the study by Ali et al. (2022).

This metric has two components for each time step: 1) $\bar{s_{t}}$, the mean network activity, and 2) $\bar{\tau_{t}}$, the mean network synaptic transmission caused by said spike activity. Both of these components were averaged across layers to ensure comparability between architectures. This measure is inversely correlated with the network's sparseness. Since the weight matrices did not differ dramatically between models, we can strongly correlate how sparse a network was with how low its energy value was (hence, how energy-efficient it was).

For *Voltage fluctuations*, we used the Scaled Median Absolute Deviation (SMAD) from 0 for the resulting membrane potentials of a batch:


$$\begin{array}{l} \mathrm{SMAD}=\frac{\mathrm{median}\left(\left|h\right|\right)}{0.67} \end{array}$$

The sampling function has a built-in shuffling property. Given the way we loop through the test set, for each iteration, we compute a Monte Carlo estimate of the expected measures. However, due to the order of magnitude of the samples, our value is statistically valid.

For |λ|*, Frequency* and *Fraction Oscillatory*, from Tables 2, 3, in the *AM+GA models* we computed all the values of *J* per trial and used them to compute the respective |λ|*, Frequency* and *Fraction Oscillatory* at each time step where neurons had spiked, and hence *J* could be computed. We 1)then averaged over those time steps, 2)then across trials for each neuron, and 3)finally averaged the values of each neuron for the per-iteration “dynamic” value. We did these computations for each layer separately. We then did the weighted average of these “dynamic” values with the “static” values (that is, independent of *J*), where the weights were defined by the average number of time steps where the network was active/silent out of the total number of time steps, respectively. The final results are then the mean and standard deviation of the per-iteration comparison. For Table 2 we also averaged the per-layer result.

We used the conventional *coefficient of variation (CV)*, where *ISI* is the interspike interval:


$$\begin{array}{l} \mathrm{CV}=\frac{\Delta \mathrm{ISI}}{\bar{\mathrm{ISI}}} \end{array}$$

### 4.6 Bound on *a* and reversal potentials

The bounds on the subthreshold adaptation constant, *a*, have a strong impact on the membrane dynamics Baronig et al., (2025). We bounded *a* ≥ 0 based on the study by (Baronig et al. 2025) and our own derivations (see “Methods” section). This regime, *a* ≥ 0, represents the adaptation regime, with higher voltages leading to greater adaptation.

The initial values for the reversal potentials were based on biological estimates, approximately matching reversal potentials in biological neurons, and were not subject to optimization. We trained 2 types of models using reversal potentials: *fixed* and *train (trainable)*. As the name suggests, for the *fixed models* we maintained the reversal threshold at our biological estimate; for the *train* models we initialized the reversal thresholds at our biological estimates but treated them as learnable parameters of the network. For each layer, all neurons shared the same reversal threshold.

## Data Availability

The original contributions presented in the study are included in the article/Supplementary material, further inquiries can be directed to the corresponding authors.
