# Supplementary material for: Conductance-based reversal potentials in spiking recurrent neural networks enhance energy efficiency and task performance
Source: Front Comput Neurosci. 2026 Jul 30;20:1870593. doi: 10.3389/fncom.2026.1870593 (PMC13468798; doi:10.3389/fncom.2026.1870593)
Supplement: Supplementary file 1 [file Data_Sheet_1.pdf]

Table 1: Table S1 - P-value matrix for *accuracy* across models (upper triangular for Arch-2L, lower for Arch-1L)

Arch-1L \ Arch-2L	Baseline	Dale	AM+GA	AMPA	GABA	AM+GA static	AMPA static	GABA static
Baseline	1	0.3259	0.001064	0.01261	0.004589	0.005965	0.02018	0.01617
Dale	0.003308	1	0.005117	0.06391	0.02894	0.02411	0.1311	0.1091
AM+GA	0.7580	$8.63 \cdot 10^{-5}$	1	0.2259	0.006092	0.9016	0.005926	0.003277
AMPA	0.6384	$9.72 \cdot 10^{-4}$	0.7289	1	0.9603	0.3783	0.3792	0.4002
GABA	0.5768	0.004666	0.6421	0.8592	1	0.2667	0.1668	0.1518
AM+GA static	0.2231	0.2284	0.2196	0.2953	0.3699	1	0.1034	0.1058
AMPA static	0.6402	0.01130	0.7396	0.9109	0.9726	0.3862	1	0.9117
GABA static	0.6130	0.001304	0.6859	0.9527	0.9008	0.3119	0.9455	1

Table 2: Table S2 - P-value matrix for *test spike energy* across models (upper triangular for Arch-2L, lower for Arch-1L)

Arch-1L \ Arch-2L	Baseline	Dale	AM+GA	AMPA	GABA	AM+GA static	AMPA static	GABA static
Baseline	1	$2.92 \cdot 10^{-6}$	$1.04 \cdot 10^{-9}$	$4.41 \cdot 10^{-7}$	$3.97 \cdot 10^{-5}$	$8.03 \cdot 10^{-8}$	$2.02 \cdot 10^{-7}$	$1.30 \cdot 10^{-8}$
Dale	$5.43 \cdot 10^{-8}$	1	$1.10 \cdot 10^{-10}$	$1.16 \cdot 10^{-4}$	0.004634	$1.63 \cdot 10^{-7}$	$1.69 \cdot 10^{-5}$	$2.08 \cdot 10^{-8}$
AM+GA	$3.80 \cdot 10^{-14}$	$4.40 \cdot 10^{-10}$	1	$9.09 \cdot 10^{-8}$	$1.37 \cdot 10^{-4}$	0.8193	$9.61 \cdot 10^{-8}$	$1.26 \cdot 10^{-7}$
AMPA	$8.71 \cdot 10^{-10}$	$2.27 \cdot 10^{-5}$	$5.39 \cdot 10^{-9}$	1	0.4663	$2.69 \cdot 10^{-4}$	0.2422	$6.97 \cdot 10^{-5}$
GABA	$1.34 \cdot 10^{-12}$	$1.02 \cdot 10^{-8}$	$1.59 \cdot 10^{-7}$	$6.29 \cdot 10^{-7}$	1	$3.05 \cdot 10^{-5}$	0.008815	0.03193
AM+GA static	$1.05 \cdot 10^{-11}$	$3.93 \cdot 10^{-9}$	0.6991	$6.24 \cdot 10^{-8}$	$1.44 \cdot 10^{-5}$	1	$2.20 \cdot 10^{-7}$	$8.16 \cdot 10^{-7}$
AMPA static	$3.49 \cdot 10^{-12}$	$1.39 \cdot 10^{-6}$	$3.56 \cdot 10^{-11}$	0.1529	$2.70 \cdot 10^{-8}$	$2.13 \cdot 10^{-5}$	1	$1.99 \cdot 10^{-4}$
GABA static	$1.07 \cdot 10^{-11}$	$1.98 \cdot 10^{-8}$	$2.54 \cdot 10^{-6}$	$1.13 \cdot 10^{-6}$	0.4882	0.01186	$1.69 \cdot 10^{-7}$	1

Table 3: Table S3 - P-value matrix for *SMAD* across models (upper triangular for Arch-2L, lower for Arch-1L)

Arch-1L \ Arch-2L	Baseline	Dale	AM+GA	AMPA	GABA	AM+GA static	AMPA static	GABA static
Baseline	1	$7.26 \cdot 10^{-4}$	$3.54 \cdot 10^{-7}$	$3.67 \cdot 10^{-5}$	$3.97 \cdot 10^{-5}$	$8.03 \cdot 10^{-8}$	$7.61 \cdot 10^{-6}$	$6.98 \cdot 10^{-6}$
Dale	$4.90 \cdot 10^{-5}$	1	$1.96 \cdot 10^{-6}$	0.002568	0.004634	$1.63 \cdot 10^{-7}$	$2.14 \cdot 10^{-4}$	$3.21 \cdot 10^{-4}$
AM+GA	$1.35 \cdot 10^{-8}$	$2.91 \cdot 10^{-7}$	1	$7.36 \cdot 10^{-4}$	$1.37 \cdot 10^{-4}$	0.8193	0.004092	$1.31 \cdot 10^{-4}$
AMPA	$3.83 \cdot 10^{-6}$	$4.17 \cdot 10^{-4}$	$1.28 \cdot 10^{-4}$	1	0.4663	$2.69 \cdot 10^{-4}$	0.1884	0.8881
GABA	$2.94 \cdot 10^{-8}$	$1.06 \cdot 10^{-6}$	0.03163	0.001515	1	$3.05 \cdot 10^{-5}$	0.04456	0.2906
AM+GA static	$6.86 \cdot 10^{-10}$	$1.38 \cdot 10^{-8}$	0.003849	$4.12 \cdot 10^{-6}$	$1.04 \cdot 10^{-4}$	1	0.001471	$1.01 \cdot 10^{-5}$
AMPA static	$2.09 \cdot 10^{-6}$	$1.10 \cdot 10^{-4}$	$9.37 \cdot 10^{-4}$	0.2204	0.01708	$2.13 \cdot 10^{-5}$	1	0.1405
GABA static	$4.66 \cdot 10^{-10}$	$1.77 \cdot 10^{-8}$	0.1606	$1.53 \cdot 10^{-5}$	0.001420	0.01186	$1.10 \cdot 10^{-4}$	1

	Parameters	AM+GA	fixed AM+GA	Dale	Baseline
1 Layer	$\lvert\lambda\rvert$	0.941 ± 0.001	0.940 ± 0.002	0.935 ± 0.001	0.937 ± 0.001
	Frequency [Hz]	7.865 ± 0.115	8.093 ± 0.313	7.770 ± 0.190	7.720 ± 0.138
	Fraction Oscillatory	0.892 ± 0.026	0.915 ± 0.010	0.845 ± 0.018	0.854 ± 0.006
2 Layers	$\lvert\lambda\rvert$	0.947 ± 0.001	0.946 ± 0.001	0.944 ± 0.001	0.945 ± 0.001
	Frequency [Hz]	7.685 ± 0.165	7.720 ± 0.218	7.653 ± 0.238	7.500 ± 0.213
	Fraction Oscillatory	0.892 ± 0.014	0.884 ± 0.013	0.870 ± 0.015	0.874 ± 0.009

Table 4: Sup material with J=0

		AM+GA		fixed AM+GA		Dale		Baseline	
	Parameters	L1	L2	L1	L2	L1	L2	L1	L2
2 Layers	$\lvert\lambda\rvert$	0.947 ± 0.002	0.947 ± 0.001	0.946 ± 0.001	0.945 ± 0.002	0.942 ± 0.001	0.945 ± 0.002	0.945 ± 0.001	0.946 ± 0.001
	Frequency [Hz]	7.268 ± 0.200	8.100 ± 0.133	7.495 ± 0.203	7.943 ± 0.230	7.748 ± 0.195	7.555 ± 0.280	7.495 ± 0.265	7.503 ± 0.160
	Fraction Oscillatory	0.889 ± 0.015	0.894 ± 0.014	0.874 ± 0.015	0.894 ± 0.012	0.868 ± 0.016	0.872 ± 0.014	0.881 ± 0.013	0.867 ± 0.005

Table 5: Sup material with J=0
